# Ipsilaterale venöse Gefäßpunktion und Katheterplatzierung nach axillärer Lymphadenektomie

**DOI:** 10.1007/s00101-026-01694-y

**Published:** 2026-06-15

**Authors:** J. Weber, F. Wappler, M. Schieren, J. Defosse

**Affiliations:** Klinik für Anästhesiologie und operative Intensivmedizin, Klinikum der Universität Witten/Herdecke, Kliniken Köln gGmbH, Ostmerheimer Str. 200, 51109 Köln, Deutschland

**Keywords:** Mammakarzinom, Lymphödem, Brustkrebsassoziiertes Lymphödem, Risikoabwägung, Periphere Venenverweilkanüle, Breast cancer, Lymphedema, Breast cancer-related lymphedema, Risk assessment, Peripheral intravenous catheter

## Abstract

Nach axillärer Lymphadenektomie (ALND) oder Radiatio bei Mammakarzinom oder malignem Melanom kann die Schädigung des lymphatischen Gewebes die Transportkapazität reduzieren und zu einer vermehrten interstitiellen Flüssigkeitsansammlung führen. Dies kann Schwellungen, Schmerzen, trophische Hautveränderungen und ein erhöhtes Infektionsrisiko verursachen. Zudem können funktionelle Einschränkungen der betroffenen Extremität psychosoziale Folgen haben. Betroffene Patienten werden üblicherweise über ihr lebenslang erhöhtes Risiko für ein Lymphödem informiert. Internationale sowie nationale Leitlinien empfehlen, ipsilaterale medizinische Eingriffe wie Gefäßpunktionen oder Blutdruckmessungen zu vermeiden. Ist eine kontralaterale Punktion schwierig, werden suboptimale Punktionsstellen wie der Fuß gewählt oder die Anlage eines zentralvenösen Katheters durchgeführt, obwohl dies mit einem erschwerten Zugang und einer eingeschränkten Visualisierung der Punktionsstelle verbunden sein kann. Frustrane Punktionsversuche können zu Schmerzen, Diskomfort und funktionellen Einschränkungen führen; insbesondere die Anlage peripherer Venenverweilkanülen an ungünstigen Stellen wie dem Fuß kann die Mobilität erheblich beeinträchtigen und das Risiko für Komplikationen wie eine tiefe Venenthrombose erhöhen. Früher war die Evidenz zu ipsilateralen Verfahren begrenzt und basierte überwiegend auf kleinen Fallstudien. Neuere Daten zeigen jedoch, dass isolierte Eingriffe wie etwa venöse Punktionen und Katheterplatzierungen nach ALND oder axillärer Strahlentherapie sicher durchgeführt werden können, sofern kein präexistierendes Lymphödem vorliegt. So sollte nach einer kontralateral frustranen Punktion eine ipsilaterale Punktion erwogen werden, bevor ungünstige Punktionsstellen gewählt oder die Anlage eines zentralen Venenkatheters erwogen wird.

## Abstract (en)

After axillary lymphadenectomy (ALND) or radiotherapy for breast cancer or malignant melanoma, damage to the lymphatic tissue can reduce the lymphatic transport capacity and increase interstitial fluid accumulation. This can lead to swelling, pain, progressive trophic skin changes and an elevated risk of infections. Moreover, functional impairment of the affected limb can result in significant psychosocial consequences. Affected patients are usually informed about their increased lifetime risk of developing lymphedema and current guidelines from numerous international and national societies recommend that ipsilateral medical interventions, such as vascular punctures for blood sampling or blood pressure measurements should be avoided. If contralateral venous access is difficult, suboptimal puncture sites such as the foot are chosen or a central venous catheter is placed, even though this is associated with limited access and reduced visualization of the puncture site. Unsuccessful venipuncture attempts can cause pain, discomfort and functional limitations; in particular, placing peripheral intravenous catheters at suboptimal sites such as the foot can substantially impair mobility and thus increase the risk of complications such as deep vein thrombosis. Until recently, evidence on ipsilateral procedures was scarce and largely derived from small case studies; however, emerging data indicate that isolated procedures such as venous puncture and catheter placement can be safely performed following ALND or axillary radiotherapy in the absence of pre-existing lymphedema. Likewise, noninvasive methods (e.g., blood pressure measurements) do not appear to elevate the risk of lymphedema, although ipsilateral interventions should remain contraindicated in patients with established lymphedema. Thus, following an unsuccessful contralateral puncture, an ipsilateral puncture should be considered before selecting unfavorable puncture sites or considering the placement of a central venous catheter.

Mit ungefähr 70.000 Neuerkrankungen in Deutschland/Jahr ist das Mammakarzinom die häufigste Krebserkrankung der Frau. Aktuellen Inzidenzraten zufolge erkrankt etwa eine von 8 Frauen im Laufe ihres Lebens an einem Mammakarzinom [21]. Im Vergleich hierzu ist das maligne Melanom mit einer Zahl von 10.000 Neuerkrankungen/Jahr in Deutschland wesentlich seltener. Hierbei kann es einerseits durch eine lymphogene Metastasierung und andererseits durch die operative und radioonkologische Therapie zu einer Schädigung lymphatischen Gewebes und konsekutiver Abnahme der lymphatischen Transportkapazität kommen. Auch die Anwendung optimal konformaler Bestrahlungsvolumina und modernster Planung der Bestrahlung kann eine Fibrosierung und Sklerosierung des lymphatischen Systems mit einem Verlust von dermalen lymphatischen Blutgefäßen und eine irreversible Schädigung des lymphatischen Gewebes nicht komplett verhindern [24]. Gemäß der International Society of Lymphology ist das Lymphödem als eine chronisch-entzündliche Erkrankung des Interstitiums als Folge einer primären oder sekundären Schädigung des Lymphdrainagesystems definiert [11]. Durch die Abnahme der lymphatischen Transportkapazität entstehen ein Ungleichgewicht des Flüssigkeitstransfers von intravasal nach interstitiell und hierdurch eine Zunahme der interstitiellen Gewebsflüssigkeit. Neben Schwellungen treten häufig auch Schmerzen und Bewegungseinschränkungen der betroffenen Extremität auf. Im Verlauf können trophische Hautveränderungen auftreten sowie Haut- und Weichteilinfektion resultieren [22]. Regelmäßige klinische Untersuchungen und eine adäquate Patienteninformation spielen in der Diagnostik und Therapie des Lymphödems eine entscheidende Rolle, da in der Anfangsphase oft keine äußerlich sichtbare Schwellung oder messbare Umfangsveränderung der betroffenen Extremität auftreten. Neben Umfangsmessungen können Sonographie, Lymphszintigraphie, Magnetresonanztomographie (MRT) und weitere radiologische Verfahren bei spezifischen Fragestellungen ergänzend eingesetzt werden. Manuelle Lymphdrainage, Kompressionstherapie, Bewegungstherapie und Hautpflege sind zentrale Bestandteile der primär konservativen Therapie beim Lymphödem [3]. Bei schweren, therapierefraktären Verläufen kann eine chirurgische Therapie, beispielsweise mittels lymphovenöser Anastomose, durchgeführt werden [3]. In einer Langzeitstudie über einen Beobachtungszeitraum von 20 Jahren konnten Petrek et al. [19] bei 49 % der Patient*innen (*n* = 263) nach Therapie eines Mammakarzinoms (Mastektomie und axilläre Lymphadenektomie [ALND]) ein Lymphödem (brustkrebsassoziiertes Lymphödem [engl. „breast cancer related lymphedema“, BCRL]) beobachten. In 77 % der Fälle (*n* = 98 von 128) trat dieses bereits innerhalb der ersten 3 Jahre nach der operativen Therapie auf. In einer Metaanalyse von Disipio et al. [5], die auf 72 Studien im Zeitraum von 2000 bis 2012 basiert, wurde eine durchschnittliche Inzidenz für das Auftreten eines BCRL von 21,4 % erhoben. Nach Selektion von Studien mit prospektivem Studiendesign (30 Studien) betrug diese noch 16,6 %. Dabei konnte das BCRL in einer Analyse von 36 Studien nahezu 4‑mal häufiger nach ALND als nach Sentinel-Lymphknotenbiopsie beobachtet werden. In Bezug auf die hohe Lebenszeitprävalenz eines Mammakarzinoms existiert daher eine relevante Anzahl an Betroffenen mit potenziell erhöhtem Risiko eines Lymphödems nach ALND oder axillärer Radiatio. Daher sind Behandler*innen damit konfrontiert, dass beispielsweise potenzielle Punktionsstellen für venöse Blutentnahmen oder die Platzierung eines peripheren Verweilkatheters nicht zur Verfügung stehen.

Vor der grundsätzlich physiologisch nachvollziehbaren Sorge vor einer zusätzlichen Belastung der potenziell reduzierten lymphatischen Transportkapazität nach ipsilateral durchgeführten medizinischen Maßnahmen (Blutdruckmessung, Platzierung einer peripheren Venenverweilkanüle [PVK] und weiteres) empfehlen internationale und nationale Fachgesellschaften, dass nach der axillären Therapie bei Mammakarzinom auf der betroffenen Seite keine medizinischen Interventionen erfolgen sollten [24]. In einer systematischen Literaturrecherche von Jakes et al. [12] wurden einerseits der Einfluss ipsilateraler medizinischer Maßnahmen auf das Risiko eines BCRL und andererseits die zum Untersuchungszeitpunkt geltenden Empfehlungen internationaler Fachgesellschaften zur Patientenaufklärung bezüglich solcher Eingriffe analysiert. Insgesamt konnten 15 Empfehlungen zu ipsilateralen medizinischen Interventionen identifiziert werden. Diese Empfehlungen raten von der Durchführung ipsilateraler medizinischer Maßnahmen ab, außer wenn diese unvermeidbar sind. Nach weiterer Selektion konnten 7 wissenschaftliche Artikel, welche den Zusammenhang von Venenpunktionen und Lymphödem nach Therapie eines Mammakarzinoms thematisierten, identifiziert werden. Diese beinhalteten eine prospektive Studie, 6 retrospektive Beobachtungsstudien und eine retrospektive Matched-Pair-Analyse. Die einzige prospektive Studie, die in die Literaturrecherche einbezogen wurde, stammt von Clark et al. [4]. Diese untersuchten die Inzidenz und den Einfluss ipsilateraler medizinischer Maßnahmen über einen Zeitraum von 3 Jahren (3, 6, 12, 24 und 36 Monate postoperativ) nach Therapie eines Mammakarzinoms. In der letzten Folgeuntersuchung, 3 Jahre postoperativ, wurde bei 20,7 % (*n* = 39) der Studienpopulation (*n* = 188) ein Lymphödem erfasst (definiert als eine neu aufgetretene Zunahme der Umfangsdifferenz zwischen ipsilateralem und kontralateralem Arm um ≥ 20 %). Innerhalb dieses Zeitraums erhielten 18 Patient*innen eine ipsilaterale Punktion (PVK: *n* = 9, einfache Venenpunktion: *n* = 6, wiederholte Messung des Blutzuckers an der Fingerbeere: *n* = 3). Bei 8 dieser Patient*innen wurde ein Lymphödem festgestellt (relatives Risiko [RR] 2,44; 95 %-Konfidenzintervall [95 %-KI]: 1,33 – 4,47). Die Autor*innen der Studie schlussfolgern, dass Venenpunktionen das Risiko eines BCRL erhöhen. Aufgrund der begrenzten Stichprobengröße ist die Generalisierbarkeit der Ergebnisse von Clark et al. [4] jedoch eingeschränkt. In den übrigen von Jakes et al. selektierten Studien fanden sich keine Hinweise auf ein erhöhtes Risiko eines BCRL nach Hautpunktionen und Platzierung eines PVK [12]. Vor dem Hintergrund dieser Studienlage empfehlen Jakes et al. ein pragmatisches Prozedere bei erhöhtem Risiko eines Lymphödems (Abb. 1).

**Abb. 1:**
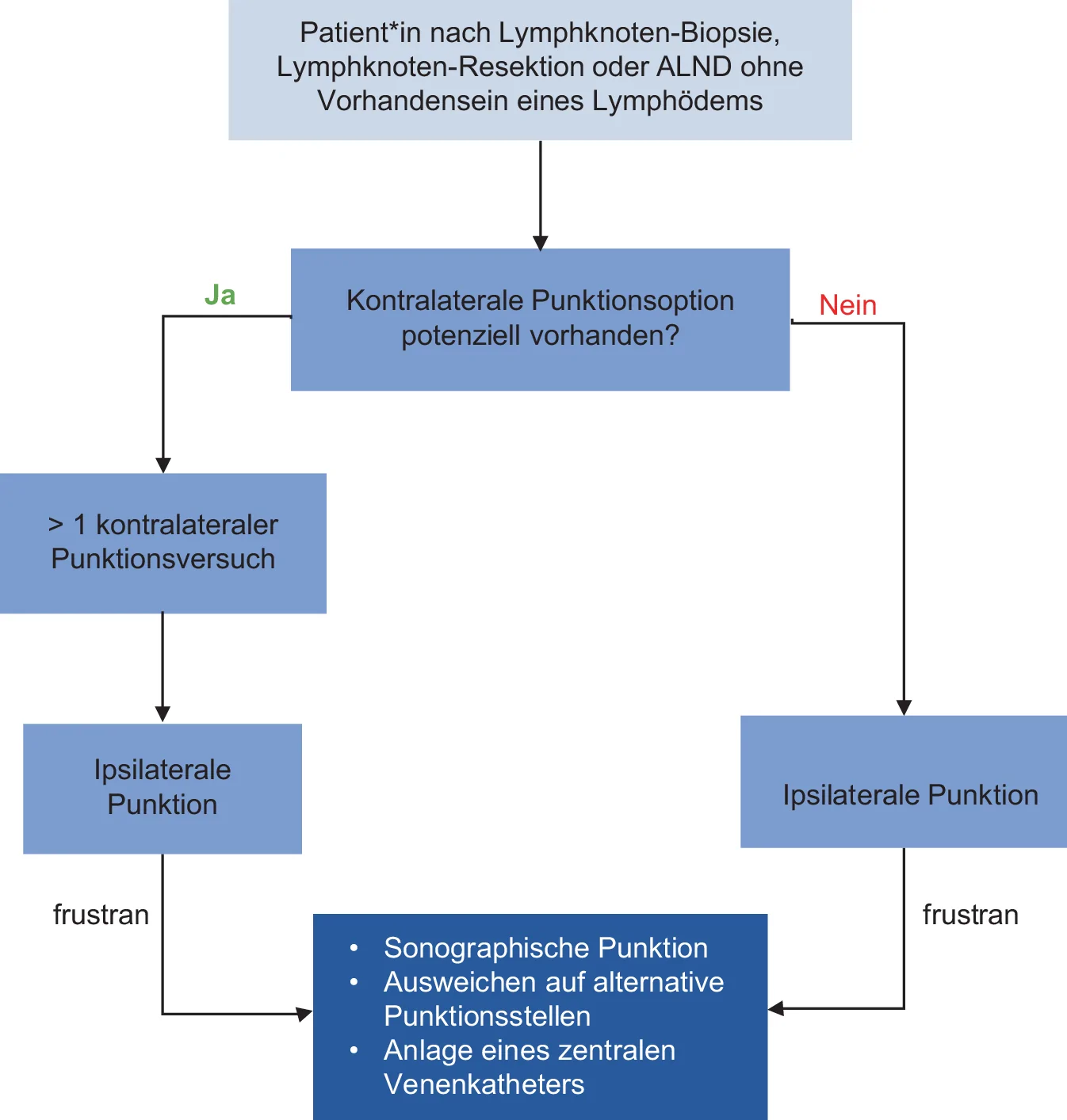
Flussdiagramm zur venösen Punktion und Katheterplatzierung bei erhöhtem Risiko eines Lymphödems, adaptiert nach Jakes et al. [8]. *ALND* Engl. axillary lymph node dissection, axilläre Lymphadenektomie.

Asdourian et al. [1] erfassten in einer Übersichtsarbeit aus dem Jahr 2016 insgesamt 31 wissenschaftliche Untersuchungen, die den Einfluss von Flugreisen, Blutdruckmessungen am ipsilateralen Arm, Hautverletzungen, Hautinfektionen und anderen Faktoren auf das Auftreten eines Lymphödems nach ALND untersuchten. Die untersuchten Studien zeigten, dass weder ipsilaterale Blutdruckmessungen noch venöse Punktionen Risikofaktoren für ein Lymphödems darstellen. Die Autor*innen der Übersichtsarbeit schlussfolgern auf Grundlage der vorliegenden Datenlage, dass die Durchführung isolierter medizinischer Maßnahmen wahrscheinlich nicht das Risiko eines BCRL, selbst nach ALND, erhöht. Als einzige prospektive Untersuchung, welche ipsilaterale Punktionen als Risikofaktor eines BCRL beobachten konnte, wird auch hier die zuvor beschriebene Untersuchung von Clark et al. genannt. Neben der geringen Fallzahl der Untersuchung wird in der Übersichtsarbeit von Asdourian et al. auch eine mögliche Verzerrung der Untersuchungsergebnisse aufgrund eines Erinnerungsbias diskutiert, da sich Patient*innen, die ein Lymphödem entwickelten, eher an zurückliegende Hautpunktionen erinnerten. Zudem wurde der zeitliche Zusammenhang zwischen der Punktion und dem Auftreten eines Ödems nicht gemessen. In diesem Kontext ist auch die im Jahr 2021 veröffentlichte retrospektive Datenerhebung von Naranjo et al. [18] hervorzuheben. Ziel dieser Untersuchung war die Analyse des Einflusses einer ipsilateralen Platzierung eines PVK auf das Auftreten eines Lymphödems nach operativer Therapie eines Mammakarzinoms. Hierzu wurden digitale Patientenakten mithilfe spezifischer Schlagworte zur einer assoziierten Komplikation untersucht. Innerhalb eines Untersuchungszeitraumes von 2 Jahren und 4 Monaten wurden 7896 Platzierungen eines PVK bei 3724 Patient*innen dokumentiert. Im Rahmen eines 2‑wöchigen postoperativen Intervalls wurden Komplikationen wie Ödeme, Kompartmentsyndrome, Infektionen und andere Ereignisse in den digitalen Patientenakten erfasst. In 2799 Fällen konnte eine ipsilaterale Platzierung eines PVK erfasst werden. In nur 2 Fällen (0,07 %) wurde eine Komplikation dokumentiert. In einem Fall kam es bei einer hochbetagten und multimorbiden Patientin 9 Jahre nach Mastektomie bei Mammakarzinom nach Operation einer Hüftfraktur und ipsilateraler Platzierung eines PVK zu einem lokal begrenzten Ödem des Ellenbogens. Im zweiten Fall trat nach einer Bandscheibenoperation der Lendenwirbelsäule eine Zunahme eines bereits zuvor bestehenden bilateralen Lymphödems der Arme nach beidseitiger Mastektomie und axillärer Radiatio bei Mammakarzinom auf. Die mediane Dauer zwischen der Therapie bei Mammakarzinom und der ipsilateralen Platzierung eines PVK betrug 1,5 Jahre. Als Limitationen dieser Untersuchung wurde das retrospektiven Studiendesign, die unerwartet niedrige Inzidenz einer Phlebitis sowie eine möglicherweise inadäquate Dokumentation in den digitalen Patientenakten angeführt.

In einer aktuellen prospektiven Studie untersuchten Ferguson et al. [8] den Einfluss einer venösen Punktion und weiterer medizinischer Maßnahmen auf das Auftreten eines BCRL bei 632 Patientinnen nach Therapie bei Mammakarzinom über einen Zeitraum von 5 Jahren. Im Rahmen der Folgeuntersuchungen wurden Umfangsmessungen der Arme und eine Befragung anhand eines standardisierten Fragebogens durchgeführt, um venöse Punktionen, Blutdruckmessungen, Traumata, Flugreisen und weitere potenziell auslösende Faktoren eines Lymphödems zu erfassen. Die Ergebnisse zeigten ebenfalls keinen Zusammenhang zwischen venösen Punktionen und dem Auftreten eines BCRL. Ein erhöhter BMI (Body-Mass-Index), eine ALND, Radiatio und das Vorhandensein einer Phlegmone waren mit einem häufigeren Auftreten von BCRL assoziiert. Eine zusätzliche prospektive Untersuchung von Kilbreath et al. [13] beobachtete 539 Patientinnen nach einer chirurgischen Therapie bei Mammakarzinom über einen Zeitraum von 18 Monaten in einem postoperativen Intervall von einem, 6, 12 und 18 Monaten. Hierbei wurden wöchentliche Befragungen durchgeführt, um Risikoereignisse wie Punktionen zu dokumentieren. Nach 18 Monaten konnte bei 46 Fällen (10, 2 %) der vollständigen Datensätze (*n* = 450) ein Lymphödem beobachtet werden. In einer Subgruppenanalyse dieser Studie war die Punktion einer peripheren Vene zur Blutentnahme ein grenzwertig signifikanter Risikofaktor eines Lymphödems (Odds Ratio [OR] 2,0; 95 %-KI: 0,8–5,2; *p* = 0,17). In der Diskussion der Studienergebnisse wird jedoch darauf hingewiesen, dass die Datenlage widersprüchliche Hinweise zu ipsilateralen venösen Punktionen liefert. Das Ergebnis der Subgruppenanalyse wurde hier aufgrund des großen Konfidenzintervalls als nichtwegweisend bewertet. Das Ergebnis der Subgruppenanalyse kann nicht ohne Weiteres auf die Gesamtpopulation übertragen werden. In der zuletzt im Mai 2017 aktualisierten S2k-Leitlinie zu Diagnostik und Therapie von Lymphödemen wird unter Bezugnahme auf ein Konsensusdokument der International Society of Lymphology darauf hingewiesen, dass medizinische Prozeduren wie Punktionen, Infusionen und die nichtinvasive Blutdruckmessung potenziell ein Lymphödem auslösen oder ein bestehendes BCRL aggravieren können. Zugleich wird die Inkonsistenz der zugrunde liegenden Datenlage hervorgehoben [2].

Eine systematische Literaturrecherche von Hadjistyllis et al. [10], die im Jahr 2023 veröffentlicht wurde, untersuchte den Einfluss von Hautpunktionen und Platzierungen eines PVK auf das Risiko eines BCRL nach ALND über einen Zeitraum von 30 Jahren (1992–2022). Insgesamt konnten hier 11 Artikel identifiziert werden, die sich mit diesem Thema befassten. In dieser Übersichtsarbeit konnte kein Anhalt für ein erhöhtes Risiko eines BCRL nach ipsilateraler Hautpunktion und Platzierung eines PVK nachgewiesen werden. Bei etwa jeder fünften Patient*in mit Mammakarzinom erfolgt eine bilaterale chirurgische Therapie, was häufig zu einer enormen Verunsicherung der Patient*innen führt – insbesondere hinsichtlich des perioperativen Verlaufs, wenn regelhaft intermittierende oder dauerhaft durchgeführte medizinische Maßnahmen an einer oder mehreren Extremitäten notwendig sind. Eine prospektive Studie von Asdourian et al. untersuchte zwischen 2013 und 2017 den Einfluss potenzieller Risikofaktoren auf das BCRL nach bilateraler chirurgischer Therapie. In dieser Studie wurden 327 Patient*innen nach chirurgischer Therapie bei bilateralem Mammakarzinom präoperativ und dann im zeitlichen Verlauf mit einer medianen Nachbeobachtungszeit von 6,1 bis 68,2 Monaten untersucht. Zur Diagnose eines Lymphödems wurde die prozentuale Volumenänderung des Arms im Vergleich zum präoperativen Ausgangswert herangezogen. Diese wurde durch die gewichtskorrigierte Volumenänderung (WAC) verwendet ($WAC=\frac{A2W1}{W2A1}-1)$, wobei A1 das präoperative und A2 das postoperative Armvolumen sowie W1 das präoperative Körpergewicht und W2 das Körpergewicht zum Zeitpunkt der Verlaufsbeobachtung darstellen [2]. In den regelmäßigen Nachsorgemaßnahmen wurde ein neu aufgetretenes Lymphödem als Zunahme der WAC ≥ 10 % im Vergleich zum präoperativen Messergebnis (bei der ersten postoperativen Nachsorgeuntersuchung nach 3 Monaten) oder zum vorherigen Messergebnis (zum zweiten Messpunkt ab dem sechsten Monat postoperativ) definiert. Zu jedem Messzeitpunkt erfolgte außerdem eine Umfrage zur Risikoerhebung (wiederholte Blutentnahmen, Injektionen, Blutdruckmessungen, Trauma, Flugreisen und weitere). In der untersuchten Kohorte (*n* = 327), die 654 gefährdete Arme umfasste, entwickelten 83 ein Lymphödem. Eine multivariate Analyse ergab, dass keiner der erfassten potenziellen Risikofaktoren signifikant mit einer erhöhten WAC in Verbindung stand. Stattdessen korrelierten hier ein BMI ≥ 25 kg/m^2^ zum Zeitpunkt der Brustkrebsdiagnose (*p* = 0,04), die ALND (*p* = 0,04) sowie eine adjuvante Chemotherapie (*p* = 0,01) signifikant mit dem Auftreten eines Lymphödems.

Die dargestellten wissenschaftlichen Erkenntnisse legen nahe, dass isolierte medizinische Maßnahmen, wie venöse Punktionen und Katheterplatzierungen, nach Therapie eines Mammakarzinoms und bei Abwesenheit eines Lymphödems ipsilateral sicher durchgeführt werden können [1, 8, 10, 12, 16]. Gleiches gilt auch für die ipsilateral durchgeführte nichtinvasive Blutdruckmessung [6, 8, 15]. In den im Jahr 2023 veröffentlichten Aktualisierungen der Leitlinie „Guideline on Monitoring during Anaesthesia“ des Australian and New Zealand College of Anaesthetists (ANZCA) werden aufgrund der Ergebnisse der aktuelleren wissenschaftlichen Untersuchungen Anpassungen der Empfehlungen bezüglich des Vorgehens nach ALND formuliert. Es wird festgestellt, dass die vorhandene Literatur keine ausreichenden Belege liefert, um die Vermeidung ipsilateraler Punktionen, Katheterplatzierungen sowie weiterer medizinischer Maßnahmen – mit Ausnahme von Fällen, in denen bereits ein Lymphödem besteht. Daraus ergibt sich die Empfehlung der ANZCA, institutionelle Richtlinien für medizinische Maßnahmen nach einer Mammakarzinom-Behandlung entsprechend anzupassen. Außerdem wird betont, dass keine allgemeine Kontraindikation für die ipsilaterale i.v.-Katheterisierung nach solch einer Therapie vorliegt. Selbst bei präexistentem BCRL (Stadien 0–3) besteht keine absolute Kontraindikation. In den Anpassungen wird daher formuliert, dass die ipsilaterale Extremität zur Messung von Vitalparametern oder für die Platzierung eines PVK verwendet werden kann [7]. Ein Expertengremium der American Society of Breast Surgeons kommt zu ähnlichen Schlussfolgerungen: Weder ipsilaterale Punktionen und Katheterplatzierungen noch nichtinvasive Blutdruckmessungen gelten nach Ansicht des Expertengremiums als kontraindiziert. Gleichzeitig weisen die meisten Experten auch darauf hin, dass ein nichtunerheblicher Teil der bestehenden Empfehlungen zur Risikovermeidung eines BCRL immer noch nicht ausreichend evidenzbasiert ist [17].

Viele Kliniken markieren oder informieren das entsprechende Patient*innenkollektiv weiter regelhaft mit Einschränkungen bei der Verwendung ihrer Extremitäten für medizinische Interventionen, was oft zu Angst und Sorge der Patient*innen sowie der Behandler*innen führt. Statt solcher allgemeiner Maßnahmen sollten Patientinnen individuell nach bevorzugten Punktionsstellen und über mögliche alternative Optionen, einschließlich ipsilateraler Punktionen, befragt und informiert werden.

Bei Patient*innen mit bestehendem ipsilateralen BCRL sollten Eingriffe an der betroffenen Extremität vermieden werden. Wiederholte frustrane kontralaterale Punktionen könnten hier dann eine zentralvenöse Katheterplatzierung begründen. Bei Vorliegen einer absoluten Kontraindikation, wie der ausdrücklichen Ablehnung durch den Patienten, spezifischen Risikofaktoren, darunter therapeutische Antikoagulation oder ein präexistentes Lymphödem des betroffenen Arms, sollten ipsilaterale Punktionen und Katheterplatzierungen vermieden werden.

Die ipsilaterale Platzierung eines PVK für mittel- oder längerfristige Infusionstherapien (mehrere Tage oder Wochen) sowie intermittierende i.v.-Arzneistoffapplikationen ist bislang unzureichend untersucht. Um mögliche Zusammenhänge zwischen diesen Maßnahmen und der Entstehung eines Lymphödems besser zu verstehen, sind weitere wissenschaftliche Untersuchungen erforderlich. Die derzeit verfügbaren Empfehlungen zur Anpassung der Verfahren bei Patientinnen nach ALND könnten im Verlauf zu einer Zunahme des betroffenen Patientinnenkollektivs führen. Dieses könnte wiederum eine Grundlage für gezielte Studien zur genannten Fragestellung bieten. Die Ergebnisse solcher Studien könnten genutzt werden, um die bestehenden Empfehlungen der involvierten Fachgesellschaften zu aktualisieren und besser an die Evidenzlage anzupassen.

Generell sollte beachtet werden, dass ipsilaterale medizinische Maßnahmen innerhalb der ersten Jahre nach Diagnosestellung, in denen ein BCRL besonders häufig auftritt, möglichst vermieden werden sollten.

Bei Patient*innen mit erhöhtem kardiovaskulären Risiko oder vor größeren operativen Eingriffen erfolgt regelhaft eine arterielle Katheterplatzierung zur invasiven Messung des arteriellen Blutdruckmessung. Im Falle einer Fehlpunktion besteht aufgrund des höheren intravasalen Drucks ein gesteigertes Risiko für die Entstehung eines Hämatoms. Daher wird in der klinischen Praxis nach Möglichkeit auf ipsilaterale arterielle Punktionen verzichtet. Generell lässt sich aufgrund der unzureichenden Datenlage keine ausreichend wissenschaftlich belegte Aussage zum Risiko von Komplikationen nach einer arteriellen Katheterisierung treffen. Eine aus dem Gebiet der interventionellen Kardiologie stammende retrospektive Analyse von Yadav et al. [21] aus 2 Kliniken (USA und Kanada) untersuchte 134 transradiale Herzkatheterverfahren bei 129 Patient*innen nach Therapie eines Mammakarzinoms. Davon wurden 42 arterielle Punktionen ipsilateral zur Seite der Brustkrebstherapie durchgeführt. Keine der Patient*innen entwickelte innerhalb von 30 Tagen oder im Langzeitverlauf von bis zu 4 Jahren ein Lymphödem, eine Phlegmone oder vaskuläre Komplikation.

Ebenso wie periphere Gefäßpunktionen stellen Regionalanästhesieverfahren an der oberen Extremität, etwa axilläre oder infraklavikuläre Plexus-brachialis-Blockaden, Standardverfahren dar. Es existieren nach aktueller Recherche auf PubMed keine systematischen Untersuchungen, die ipsilaterale Plexus-brachialis-Blockaden oder andere Regionalanästhesieverfahren bei Patient*innen nach ALND oder Mammachirurgie hinsichtlich BCRL- oder anderen Komplikationen untersuchten. Die oben dargestellte Literatur zeigt, dass dominante Risikofaktoren für das Auftreten eines BCRL eine ALND, Radiatio, BMI und Infektionen darstellen [9, 14]. Vor diesem Hintergrund lässt sich aus der verfügbaren Evidenz keine spezifische Kontraindikation für ipsilaterale Regionalanästhesieverfahren ableiten. Vielmehr heben Reviews und Leitlinien hervor, dass pauschale Verbote einzelner Maßnahmen an der betroffenen Extremität nicht hinreichend evidenzbasiert sind [1, 20].

Zudem ist zwischen einer kurzzeitig liegenden PVK im Rahmen ambulanter Eingriffe und einer mehrtägigen stationären Infusionstherapie klar zu unterscheiden. Für die Frage, ob die Liegedauer einer PVK an der ipsilateralen oberen Extremität das Risiko eines BCRL beeinflusst, existieren bislang keine belastbaren Daten; keine der verfügbaren Studien untersucht diese Fragestellung. Gut belegt ist jedoch, dass eine verlängerte Verweildauer das Risiko sowohl für eine Extravasation als auch für katheterassoziierte Infektionen erhöht. Mehrere große Studien zeigen, dass PVK-Versagen – häufig bedingt durch Infiltration oder Extravasation – mit zunehmender Liegedauer signifikant häufiger auftritt [23]. Daher sollte eine ipsilaterale Katheterplatzierung für mehrtägige Infusionstherapien sorgfältig abgewogen und nach Möglichkeit auf alternative Punktionsstellen ausgewichen werden.

Insgesamt unterstreicht die aktuelle Evidenz den Bedarf an prospektiven Studien, die das Risiko von Lymphödem- und Gefäßkomplikationen nach ipsilateralen arteriellen Gefäßpunktionen, Regionalanästhesieverfahren sowie einer mittel- und längerfristigen PVK-Liegedauer systematisch erfassen, um die bislang überwiegend erfahrungsbasierten Empfehlungen weiter zu präzisieren (Tab. 1).

**Tab. 1:** Übersicht über die dargestellten Kernaussagen. *BCRL* Engl. breast cancer related lymphedema, Brustkrebs-assoziiertes Lymphödem; *NIBP* Engl. non-invasive blood pressure measurement, nicht-invasive Blutdruckmessung; *PVK* peripherer Venenverweilkatheter.

Maßnahme	Evidenzlage	Kernaussage	Literaturstelle
NIBP	Prospektive Kohortenstudien (*n* > 600)	Kein Zusammenhang zwischen ipsilateraler NIBP und Entstehung eines BCRL	[1, 8]
PVK	Retrospektive Großkohortenstudie (*n* = 3724, 7896 PVK-Platzierungen), systematische Reviews	Insgesamt geringe Komplikationsrate (0,07 %); kein Hinweis auf Assoziation mit dem BCRL	[10, 12, 18]
Venöse Punktionen allgemein	Prospektive Kohortenstudie	Keine signifikante Assoziation	[1, 8, 12, 13]
Punktion arterieller Gefäße	Retrospektive 2‑Zentren-Studie (42 ipsilaterale Gefäßpunktionen)	Keine Komplikationen nach dem Verfahren und im Follow-up nach 30 Tagen und bis zu 4 Jahren	[25]

## Fazit für die Praxis

- Nach axillärer Lymphadenektomie (ALND) oder Radiatio besteht ein lebenslang erhöhtes Risiko für die Entwicklung eines Lymphödems.
- Patient*inneninformationen enthalten oft allgemeine Verbote zur Durchführung ipsilateraler medizinischer Maßnahmen. Frühere wissenschaftliche Daten zur Sicherheit ipsilateraler Punktionen und Katheterplatzierungen waren begrenzt und basierten überwiegend auf Fallstudien, doch aktuelle Untersuchungen zeigen, dass isolierte medizinische Maßnahmen unter bestimmten Bedingungen sicher durchgeführt werden können.
- Statt eines generellen Verbots ipsilateraler Maßnahmen sollten die Auswahl der Stelle der Maßnahme vor dem Hintergrund einer individuellen Risiko-Nutzen-Abwägung sowie Diskussion alternativer Optionen evaluiert werden.
- Bei präexistentem Lymphödem sollten ipsilaterale medizinische Maßnahmen auch weiterhin vermieden werden.
- Weitere Studien sind erforderlich, um die langfristigen Auswirkungen ipsilateraler medizinischer Maßnahmen, wie perioperativer venöser Infusionstherapie und Arzneistoffapplikation, auf die Entstehung eines Lymphödems präziser zu untersuchen.

## Data Availability

Alle dieser Arbeit zugrunde liegenden Daten sind in diesem Artikel enthalten.

## References

[1] Asdourian MS, Skolny MN, Brunelle C et al (2016) Precautions for breast cancer-related lymphoedema: risk from air travel, ipsilateral arm blood pressure measurements, skin puncture, extreme temperatures, and cellulitis. Lancet Oncol 17:e392–e405 27599144 10.1016/S1470-2045(16)30204-2

[2] Asdourian MS, Swaroop MN, Sayegh HE et al (2017) Association Between Precautionary Behaviors and Breast Cancer-Related Lymphedema in Patients Undergoing Bilateral Surgery. J Clin Oncol 35:3934–3941 28976793 10.1200/JCO.2017.73.7494 PMC5721227

[3] Chang DW, Dayan J, Greene AK et al (2021) Surgical Treatment of Lymphedema: A Systematic Review and Meta-Analysis of Controlled Trials. Results of a Consensus Conference. Plast Reconstr Surg 147:975–993 33761519 10.1097/PRS.0000000000007783

[4] Clark B, Sitzia J, Harlow W (2005) Incidence and risk of arm oedema following treatment for breast cancer: a three-year follow-up study. QJM 98:343–348 15820971 10.1093/qjmed/hci053

[5] Disipio T, Rye S, Newman B et al (2013) Incidence of unilateral arm lymphoedema after breast cancer: a systematic review and meta-analysis. Lancet Oncol 14:500–515 23540561 10.1016/S1470-2045(13)70076-7

[6] Dixon JM, Elder K, Mclaughlin S (2019) Evidence-Based Advice for Patients Following Axillary Surgery. Breast Cancer Manag 8:bmt-2018-0018

[7] Eley V, Bradley S, Bradley P (2023) PG18(A) Guideline on monitoring during anaesthesia 2017 Appendix 1 2023, ANZCA: https://www.anzca.edu.au/getContentAsset/6296ef66-b43b-4dc6-938b-770f94ba7e6e/80feb437-d24d-46b8-a858-4a2a28b9b970/PG18(A)-Guideline-on-monitoring-during-anaesthesia-2017.PDF?language=en&view=1, abgerufen am 15.01.2026

[8] Ferguson CM, Swaroop MN, Horick N et al (2016) Impact of Ipsilateral Blood Draws, Injections, Blood Pressure Measurements, and Air Travel on the Risk of Lymphedema for Patients Treated for Breast Cancer. J Clin Oncol 34:691–698 26644530 10.1200/JCO.2015.61.5948 PMC4872021

[9] Gillespie TC, Sayegh HE, Brunelle CL et al (2018) Breast cancer-related lymphedema: risk factors, precautionary measures, and treatments. Gland Surg 7:379–403 30175055 10.21037/gs.2017.11.04 PMC6107585

[10] Hadjistyllis M, Soni A, Hunter-Smith DJ et al (2024) A systematic review of the complications of skin puncturing procedures in the upper limbs of patients that have undergone procedures on the axilla or breast. Ann Transl Med 12:70 39118962 10.21037/atm-23-1400 PMC11304417

[11] International Society of Lymphology (2013) The diagnosis and treatment of peripheral lymphedema: 2013 Consensus Document of the International Society of Lymphology. Lymphology 46:1–11 23930436

[12] Jakes AD, Twelves C (2015) Breast cancer-related lymphoedema and venepuncture: a review and evidence-based recommendations. Breast Cancer Res Treat 154:455–461 26589315 10.1007/s10549-015-3639-1

[13] Kilbreath SL, Refshauge KM, Beith JM et al (2016) Risk factors for lymphoedema in women with breast cancer: A large prospective cohort. Breast 28:29–36 27183497 10.1016/j.breast.2016.04.011

[14] Martinez-Jaimez P, Armora Verdu M, Forero CG et al (2022) Breast cancer-related lymphoedema: Risk factors and prediction model. J Adv Nurs 78:765–775 34363640 10.1111/jan.15005

[15] Mclaughlin SA (2012) Lymphedema: separating fact from fiction. Oncology (Williston Park) 26:242–249 22545305

[16] Mclaughlin SA, Wright MJ, Morris KT et al (2008) Prevalence of Lymphedema in Women With Breast Cancer 5 Years After Sentinel Lymph Node Biopsy or Axillary Dissection: Objective Measurements. J Clin Oncol 26:5213–5219 18838709 10.1200/JCO.2008.16.3725 PMC2652091

[17] Mclaughlin SA, Desnyder SM, Klimberg S et al (2017) Considerations for Clinicians in the Diagnosis, Prevention, and Treatment of Breast Cancer-Related Lymphedema, Recommendations from an Expert Panel: Part 2: Preventive and Therapeutic Options. Ann Surg Oncol 24:2827–2835 28766218 10.1245/s10434-017-5964-6

[18] Naranjo J, Portner ER, Jakub JW et al (2021) Ipsilateral Intravenous Catheter Placement in Breast Cancer Surgery Patients. Anesth Analg 133:707–712 34043309 10.1213/ANE.0000000000005597

[19] Petrek JA, Senie RT, Peters M et al (2001) Lymphedema in a cohort of breast carcinoma survivors 20 years after diagnosis. Cancer 92:1368–1377 11745212 10.1002/1097-0142(20010915)92:6<1368::aid-cncr1459>3.0.co;2-9

[20] Reztsova M, Walker JC (2025) Breast Cancer-Related Lymphedema Risk Factors and Precautionary Behaviors. Curr Breast Cancer Rep 17:1-10

[21] Robert Koch-Institut, Gesellschaft Der Epidemiologischen Krebsregister in Eutschland E.V. (2023) Krebs in Deutschland für 2019/2020, 14. Ausgabe, Robert Koch-Institut, Berlin, https://www.krebsdaten.de/Krebs/DE/Content/Publikationen/Krebs_in_Deutschland/krebs_in_deutschland_node.html, abgerufen am 15.01.2026

[22] Shih YC, Xu Y, Cormier JN et al (2009) Incidence, treatment costs, and complications of lymphedema after breast cancer among women of working age: a 2‑year follow-up study. J Clin Oncol 27:2007–2014 19289624 10.1200/JCO.2008.18.3517

[23] Webster J, Osborne S, Rickard CM et al (2019) Clinically-indicated replacement versus routine replacement of peripheral venous catheters. Cochrane Database Syst Rev 1:CD007798 30671926 10.1002/14651858.CD007798.pub5 PMC6353131

[24] Wilting J, Bartkowski R, Baumeister R et al (2017) S2k-Leitlinie: Diagnostik und Therapie der Lymphödeme. AWMF-Reg.-Nr. 058-001. https://register.awmf.org/de/leitlinien/detail/058-001wobei A1 das präoperative und A2 das postoperative Armvolumen sowie W1 das präoperative Körpergewicht und W2 das Körpergewicht zum Zeitpunkt der Verlaufsbeobachtung darstellen [2]., abgerufen am 15.01.2026

[25] Yadav PK, Bagur R, Baquero GA et al (2015) Safety and Feasibility of Transradial Catheterization in Breast Cancer Survivors: A 2‑Center International Experience. JACC Cardiovasc Interv 8:639–641 25907093 10.1016/j.jcin.2014.08.018

